# A water solubility prediction algorithm based on the StackBoost model

**DOI:** 10.1371/journal.pone.0330598

**Published:** 2025-08-29

**Authors:** Bin Pan, Xiaoyu Hou, Mingxin Zhang, Jingxian Yu, Conghui Zhang, Yunhui Zhang, Xiaolong Su, Shuangcai Li

**Affiliations:** 1 College of Science, LiaoNing Petrochemical University, Fushun, China; 2 School of Artificial Intelligence and Software, LiaoNing Petrochemical University, Fushun, China; UCSI University Kuala Lumpur Campus: UCSI University, MALAYSIA

## Abstract

Aqueous solubility, an essential physical property of compounds, has significant applications across various fields. However, verifying the solubility of compounds through experimental methods often requires substantial human and material resources. To address this issue, this study introduces the StackBoost model for predicting the solubility of organic compounds and systematically compares it with five well-known ensemble learning algorithms: Adaptive Boosting (AdaBoost), Gradient Boosted Regression Trees (GBRT), Light Gradient Boosting Machine (LGBM), Extreme Gradient Boosting (XGBoost), and Random Forest (RF). The prediction results indicate that the StackBoost model excels in predicting aqueous solubility, achieving a coefficient of determination (${\text{R}}^{2}$) of 0.90, a root mean square error (RMSE) of 0.29, and a mean absolute error (MAE) of 0.22, significantly outperforming the other comparative models. Furthermore, this study further conducted high-throughput screening on large-scale datasets and successfully identified compounds with high potential for water solubility. Additionally, the model’s generalization ability is verified through transfer learning. Although the performance of the StackBoost model decreases when applied to different datasets, it still shows considerable transferability, making it a more generalizable prediction model for aqueous solubility.

## Introduction

Water solubility is a crucial property of compounds, holding significant value in various fields such as geochemistry, climate prediction, biochemistry, drug design, agrochemical design, and protein-ligand binding [1]. For instance, in the case of pesticide compounds, poor water solubility can lead to precipitation from screening buffers, resulting in erroneous outcomes, false leads, and high risks of increased costs and formulation challenges during practical development. In recent years, researchers have begun to utilize machine learning to address the prediction of chemical solubility properties, aiming to develop compounds with rapid and efficient solubility [2–5].

With the rapid development of machine learning algorithms and their software implementations, ensemble learning has gained widespread attention due to its robust performance on multi-task cheminformatics datasets.Yang et al. employed the Support Vector Regression (SVR) method, combined with computed molecular descriptors, to establish prediction models for several physicochemical properties of alkylbenzene compounds. By identifying the optimal hyperplane in a high-dimensional feature space, they effectively captured the complex relationship between molecular structure and water solubility [6]. Fioressi et al. predicted the water solubility of 1,211 approved heterogeneous pesticide compounds using a widely applicable multiple linear regression (MLR) model, incorporating molecular descriptors such as topological, fingerprint, and flexibility descriptors. By exploring approximately 18,000 structural variables and utilizing the Replacement Method to select the most representative descriptors, they achieved optimal predictive performance [7]. Kherouf et al. developed prediction models for the water solubility of 68 phenolic compounds using both MLR and Artificial Neural Networks (ANN) methods, combined with computed three-dimensional structural molecular descriptors. After selecting descriptors through a Genetic Algorithm (GA), the ANN model demonstrated superior prediction accuracy compared to MLR [8]. Currently, ensemble learning methods are increasingly becoming an effective approach to enhance prediction accuracy.

Traditional machine learning models, such as LGBM and XGBoost, typically perform well on training data, but they are prone to overfitting when faced with complex or high-noise data. This means that the model overly relies on noise and local features in the training data, leading to a decrease in its predictive ability on new data. To address this issue, ensemble learning methods have been widely applied. Ensemble learning combines the predictions of multiple base learners, effectively reducing the bias and variance of individual models, thereby improving the model’s generalization ability and stability. Dietterich et al. used ensemble learning methods to combine the predictions of multiple base learners, establishing an effective multi-classifier system, which enhanced the stability and prediction accuracy of the model [9]. To further improve the model’s performance, Snoek et al. employed Bayesian optimization, combined with hyperparameter tuning for machine learning algorithms, to address the overfitting problem and significantly enhance the model’s predictive performance [10]. Rasmussen and Williams, on the other hand, used Gaussian process models combined with Bayesian inference methods, successfully solving the generalization problem of traditional models and improving the model’s robustness [11,12].

In recent years, ensemble learning has made significant progress in the field of cheminformatics, especially in molecular property prediction tasks. The AdaBoost algorithm proposed by Li and Zhang improves model performance in imbalanced data classification tasks by adjusting the sample weighting strategy, effectively enhancing its ability to represent complex nonlinear relationships [13]. Additionally, Zhang et al.’s improvement to the AdaBoost algorithm, by adjusting the weighting strategy of weak learners, significantly increased its accuracy in fraud detection tasks [14]. The Extreme Gradient Boosting (XGBoost) algorithm proposed by Chen and Guestrin introduced second-order gradient information, column sampling, and regularization strategies, significantly alleviating overfitting issues [15]. In recent years, researchers have made numerous optimizations to XGBoost to improve its performance on specific tasks. Huang et al. (2022) applied XGBoost to the prediction of the COVID-19 pandemic in the United States, and the results showed that it outperformed the traditional ARIMA model in prediction accuracy [16]. The LightGBM algorithm, proposed by Ke and Yang, improves the training speed and memory efficiency of large-scale high-dimensional data through gradient-based one-sided sampling (GOSS) and exclusive feature bundling (EFB) [17]. Recent research has further improved the performance of LightGBM. Wang et al. proposed an improved LightGBM hybrid ensemble model, incorporating the whale optimization algorithm (WOA) for hyperparameter optimization and using Jacobian regularization to reduce noise [18].

Although the aforementioned ensemble learning algorithms perform well in molecular property prediction, existing research still predominantly uses single algorithms or simple Bagging/Voting ensemble methods, lacking in-depth exploration of the internal complementarity of gradient boosting models. Furthermore, current research faces the following issues: first, the diversity and size of datasets are insufficient, which prevents them from fully representing a variety of experimental types, thus affecting the model’s generalization ability; second, traditional machine learning regression methods tend to overfit when faced with complex datasets, leading to poor predictive performance; third, traditional machine learning methods struggle with high-dimensional feature spaces, where redundant or irrelevant features can affect the model’s predictive accuracy. Therefore, this paper proposes a new ensemble learning framework, StackBoost, which combines the strengths of LGBM and XGBoost, aiming to improve the model’s prediction performance and stability.

Therefore, this study constructs a new ensemble model, StackBoost, based on the solubility dataset of A-I compounds by stacking the two base models, LGBM and XGBoost. The outputs of these models are used as new features input into the GBRT meta-learner. StackBoost not only fully leverages the strengths of each base learner but also effectively mitigates overfitting through the stacking strategy. To further optimize the model’s performance and reduce the risk of overfitting, Optuna is employed for hyperparameter tuning. Additionally, to validate the stability of the StackBoost model, Bayesian models are used to verify its prediction results, ensuring the model’s reliability and robustness across different datasets. This research evaluates the model’s performance using three widely used regression evaluation metrics: ${\text{R}}^{2}$, RMSE, and MAE, and conducts a systematic comparison with five mainstream ensemble learning algorithms, including AdaBoost, GBRT, LGBM, XGBoost, and RF.

## Dataset and preprocessing

### Dataset description

The experimental data of water solubility in this study were obtained from the AqSo1DB database, which is an open-access and well-structured compound database [13]. This study collected nine water solubility datasets, encompassing 9,982 compounds with water solubility data. Using topological and physicochemical two-dimensional descriptors from the datasets, all Chemical Abstracts Service(CAS) and Sybyl Line Notation(SLN) identifiers were converted into Simplified Molecular Input Line Entry System(SMILES) and validated. The nine datasets were standardized and merged into a single dataset [14–16].

The data are shown in Table 1. Dataset A is derived from the open-source chemical property database eChemPortal; after filtering by temperature range, 3,656 valid compounds were retained out of 6,110 compounds. Dataset B comes from the EPISuite Data website; through temperature filtering and SMILES validation, 3,542 valid instances were retained from 4,651 compounds. Dataset C originates from the study by Raevsky et al., containing solubility data for 1,325 solid compounds along with their SMILES representations. Dataset D also comes from the EPISuite Data website, retaining 163 liquid and crystalline compounds. Dataset E is taken from the AQUASOL and PHYSPROP databases, including 249 solubility values and SMILES data. Dataset F is sourced from the study by Wang et al.; after converting SLN to SMILES, 798 valid instances were obtained from 1,210 compounds. Dataset G includes solubility data measured at 25^°^C for 1,144 compounds, ultimately retaining 139 valid compounds. Dataset H is from Wang et al.’s cleaning of datasets used by Jain and Yalkowsky, containing 322 liquid compounds and 256 solid compounds, with 64 valid compounds retained in the end. Dataset I contains 94 compounds, with 46 valid compounds retained [17–26]. Detailed information is shown in Table 1:

**Table 1:** List of datasets in AqSolDB.

Dataset ID	Original Size	Filtered Size	Compound Representations	Solubility Units
A	6110	3656	name, CAS	g/L, mg/L, μg/L
B	4651	3542	name, CAS	LogS
C	2603	1325	name, SMILES	LogS
D	2115	163	name, CAS	LogS
E	1291	249	name, SMILES, CAS	LogS
F	1210	798	SLN	LogS
G	1144	139	name, SMILES	LogS
H	578	64	SLN	LogS
I	94	46	name, SMILES, InChI	μM

In this study, the AqSolDB dataset, which includes multiple physicochemical features, was utilized for solubility prediction research [27–32]. The ID serves as the unique identifier for compounds, distinguishing data from different sources; Name refers to the name of the compound; InChI is the IUPAC International Chemical Identifier for the compound; InChIKey is the hashed form of InChI, simplifying the unique identification of chemical substances; SMILES is a linear notation used to represent the molecular structure of chemicals; the Solubility column represents the experimental water solubility values; SD (Standard Deviation) quantifies the dispersion of multiple measurement results; Occurrences indicates the frequency of the compound in the dataset; Group is a label representing the quality of the data. The physicochemical properties include Mol Wt (Molecular Weight), Mol LogP (Molecular LogP), and Mol MR (Molecular Refractivity). Molecular structure descriptors include Heavy Atom Count, Num H Acceptors (Number of Hydrogen Bond Acceptors), Num H Donors (Number of Hydrogen Bond Donors), and Num Heteroatoms (Number of Heteroatoms). Num Rotatable Bonds represent the number of rotatable bonds in the compound. Electronic and ring structure features include Num Valence Electrons, Num Aromatic Rings, and Num Saturated Rings. Topological and physicochemical features include TPSA (Topological Polar Surface Area), Labute ASA (Labute’s Approximate Surface Area), Balaban J, and Bertz CT. These multiple features contribute to the accurate prediction of compound solubility in machine-learning models [33–36]. Among them, Solubility is the target feature of the model, and the other 25 features serve as input features. As shown in Table 2:

**Table 2:** List of available information in each column of AqSolDB.

Column Name	Description	Type
ID	ID from source (also shows the source)	string
Name	Name of compound	string
InChI	The IUPAC International Chemical Identifier	string
InChIKey	Hashed form of InChI value	string
SMILES	SMILES representation of compound	string
Solubility	Experimental aqueous solubility value (LogS)	float
SD	The standard deviation of multiple occurrences	float
Occurrences	Number of occurrences of compound	integer
Group	Generated reliability group (G1, G2, G3, G4, G5)	string
Mol Wt	Molecular weight	float
Mol LogP	Octanol-water partition coefficient	float
Mol MR	Molar refractivity	float
Heavy Atom Count	Number of H-atoms	integer
Num H Acceptors	Number of acceptors	integer
Num H Donors	Number of H donors	integer
Num Heteroatoms	Number of atoms not carbon or hydrogen	integer
Num Rotatable Bonds	Number of rotatable bonds	integer
Num Valence Electrons	Number of valence electrons	integer
Num Aromatic Rings	Number of aromatic rings	integer
Num Saturated Rings	Number of saturated rings	integer
Num Aliphatic Rings	Number of aliphatic rings	integer
Ring Count	Number of total rings	integer
TPSA	Topological polar surface area	float
Labute ASA	Description	float
Balaban J	ID from source (also shows the source)	float
Bertz CT	Name of compound	float

### Data preprocessing

To ensure consistency across different units, the solubility values of various compounds were converted to mol/L, and the InChI notation was used for validation. For non-numerical features, the One-Hot Encoding method was employed to transform them, avoiding computational bias caused by differences in feature scales. The data were normalized to highlight weaker features and make the data more closely resemble a standard normal distribution, thereby improving the accuracy and convergence speed of the algorithm. Finally, the Singular Value Decomposition (SVD) dimensionality reduction technique was applied to reduce computational complexity and enhance the model’s accuracy and stability.

**Fig 1:**
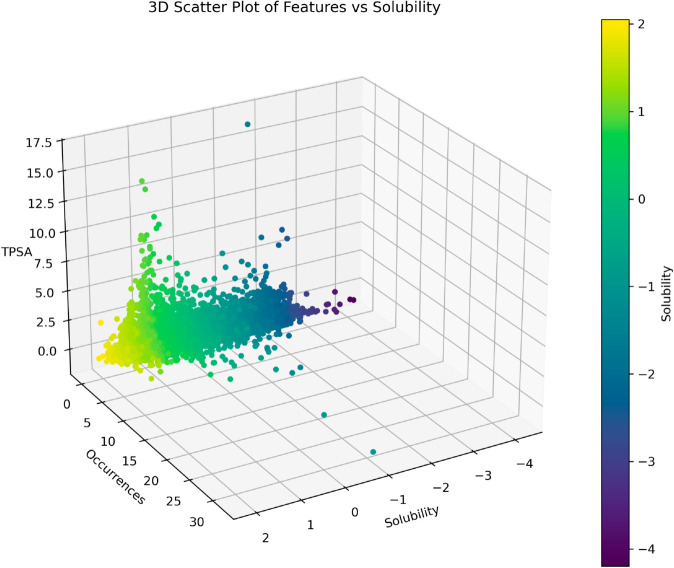
The scatter plot of the target variable solubility dataset.

To more intuitively display the distribution of the target variable, this paper visualizes Solubility using a scatter plot, as shown in Fig 1. From the figure, it can be seen that the range of solubility values in the dataset spans from -4 to 2, with most data points concentrated in the lower solubility intervals. In particular, data points exhibit a high density in the range from -4 to -2, indicating that solubility values within this range occur more frequently in the dataset. As solubility increases, the data points gradually become sparse, suggesting that compounds with higher solubility are relatively fewer. Additionally, the lower solubility values (deep purple regions) in the figure correspond to lower occurrences and TPSA values. In the higher solubility intervals, the data points show a more dispersed trend, indicating a sparser distribution of compounds in this range. Through the color gradient, it can be further observed that compounds with lower solubility (deep purple regions) are more common in the dataset, while as solubility increases, the number of compounds significantly decreases and their distribution becomes more scattered. The nonlinear distribution characteristic of solubility indicates that compounds with lower solubility dominate the dataset, whereas those with higher solubility are relatively scarce.

## Application of the model

### Machine learning regression model

**Fig 2:**
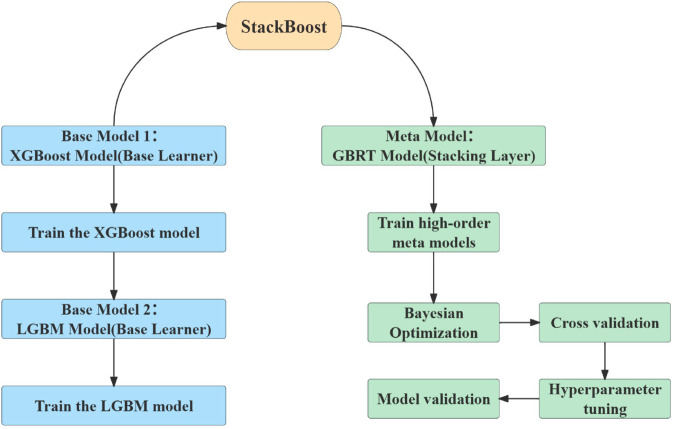
Schematic workflow diagram of the StackBoost model framework.

Fig 2 illustrates the overall workflow of the StackBoost model. The StackBoost model utilizes base models such as XGBoost and LGBM to make predictions on the data. These predictions are used as inputs to train a GBRT meta-model for the final prediction. During the model training process, StackBoost employs Bayesian optimization techniques to fine-tune the hyperparameters of the GBRT meta-model, while incorporating cross-validation strategies to ensure the model’s stability and robustness across different datasets. By stacking multiple models, the StackBoost model effectively enhances its prediction accuracy and generalization ability when handling complex data. Additionally, the StackBoost model uses Optuna for hyperparameter optimization. The preprocessed data is then used for training and prediction, with the dataset randomly split into training and testing sets at a ratio of 80% to 20%.

**Fig 3:**
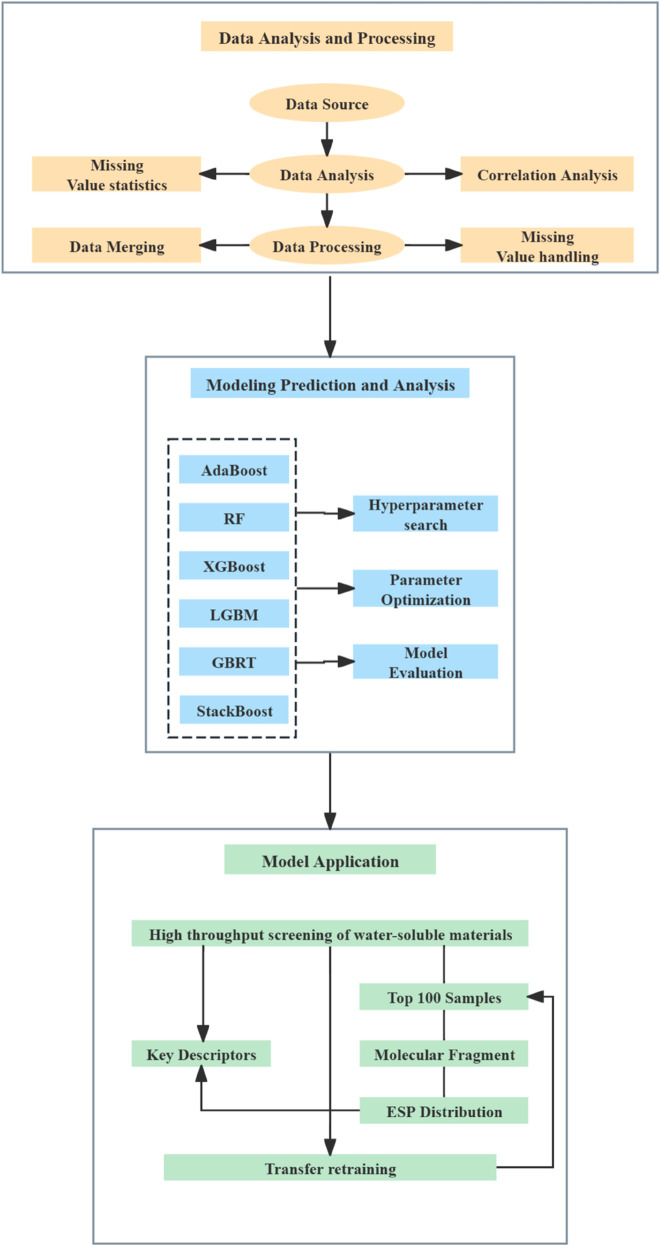
Workflow diagram of the prediction algorithm framework.

To improve the accuracy and generalization ability of water solubility prediction for materials, a multi-level, multi-dimensional StackBoost model was constructed. The overall workflow of this study is illustrated in Fig 3, which includes three parts: data analysis and processing, model prediction and analysis, and model application. For comparison, the StackBoost model was systematically compared with five mainstream ensemble learning algorithms: AdaBoost, GBRT, LGBM, XGBoost, and RF. A total of 8,352 data samples were used as the training set. The six trained ensemble learning models were employed to predict and systematically compare the water solubility of materials. Subsequently, the trained StackBoost model was applied to conduct high-throughput screening on the dataset.

### Evaluation metrics

This paper primarily utilizes ${\text{R}}^{2}$, RMSE, and MAE as metrics to evaluate and assess the model results. These three indicators quantitatively measure the discrepancies between the model’s predictions and the actual values. The calculation formula for ${\text{R}}^{2}$ is as follows:

$$R^{2}=1-\frac{SS_{\text{res}}}{SS_{\text{tot}}}$$ (1)

${\text{R}}^{2}$ serves as a critical quantitative measure for assessing the fit of regression models, with values ranging from 0 to 1. A value closer to 1 indicates a stronger fit of the model, whereas a value closer to 0 indicates a poorer fit. SSres, the sum of squared residuals, represents the squared differences between predicted values and actual values; SStot, the total sum of squares, denotes the squared differences between actual values and their mean. The calculation formula for RMSE is as follows:

$$RMSE=\sqrt{\frac{1}{n}\sum_{i=1}^{n}(y_{i}-{\hat{y}}_{i}{)}^{2}}$$ (2)

In this context, *n* represents the number of samples, *y*_*i*_ signifies the actual value of the *i*-th sample, and ${\hat{y}}_{i}$ signifies the predicted value of the *i*-th sample. RMSE measures the square root of the differences between predicted and actual values; lower RMSE values indicate better model accuracy. Another metric, the coefficient of determination *R*^2^, serves as a key quantitative standard for evaluating the fit of regression models. Values range from 0 to 1, with closer to 1 indicating a stronger fit and closer to 0 indicating a poorer fit.

$$MAE=\frac{1}{n}\sum_{i=1}^{n}|y_{i}-{\hat{y}}_{i}|$$ (3)

In this context, *n* represents the number of samples, while *y*_*i*_ and ${\hat{y}}_{i}$ denote the actual and predicted values for the *i*-th sample, respectively. MAE measures the average absolute deviation between predicted and actual values, indicating the mean degree to which the prediction model deviates from actual observations. A lower MAE indicates better predictive accuracy of the model.

## Results and discussion

To verify the impact of increasing the sample size on training and prediction results, first, 70% of the 9,936 data samples from eight datasets (A-H) were randomly selected as the training set, with the remaining 30% as the test set. Using 6,955 data samples, the StackBoost model was trained, achieving an *R*^2^ value of 0.857 on the test set. Subsequently, 80% of the 9,936 samples were randomly selected as the training set, and 20% as the test set. Using 7,949 data samples to train the StackBoost model resulted in an *R*^2^ value of 0.90 on the test set. With the increase in training samples, the *R*^2^ value improved by 0.043. Finally, 90% of the 9,936 samples were randomly selected as the training set, and 10% as the test set. Using 8,942 samples to train the StackBoost model, the test set *R*^2^ value remained at 0.90. As the number of training samples increased, the improvement in model performance tended to saturate. Therefore, this method constructs a model with high predictive accuracy using a relatively small dataset.

Table 3 presents the performance metrics of AdaBoost, GBRT, LGBM, XGBoost, RF, and StackBoost models in predicting solubility.

**Table 3:** Assessment values of machine learning models.

Model	$R^{2}$	RMSE	MAE
AdaBoost	0.67	0.57	0.44
GBDT	0.84	0.40	0.28
LGBM	0.79	0.46	0.33
XGBoost	0.83	0.40	0.29
RF	0.80	0.44	0.30
StackBoost	0.90	0.29	0.22

**Fig 4:**
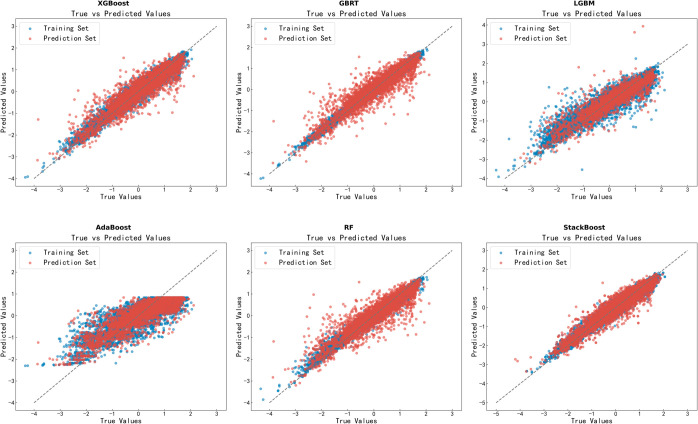
Comparison of training and predicted values of compound water solubility for different machine learning models: (A) AdaBoost, (B) GBDT, (C) LGBM, (D) RF, (E) XGBoost, (F) RF, and (G) StackBoost.

The AdaBoost model has an ${\text{R}}^{2}$ of 0.67, RMSE of 0.57, and MAE of 0.44, indicating larger prediction errors and weaker performance on complex data. The GBRT model shows improved results with an ${\text{R}}^{2}$ of 0.84, RMSE of 0.40, and MAE of 0.28, performing better compared to AdaBoost.The LGBM model achieves an ${\text{R}}^{2}$ of 0.79, RMSE of 0.46, and MAE of 0.33, indicating it performs slightly less effectively than GBRT but still delivers strong results on large datasets. The XGBoost model has an ${\text{R}}^{2}$ of 0.83, RMSE of 0.40, and MAE of 0.29, demonstrating strong predictive capabilities on complex data. The RF model records an ${\text{R}}^{2}$ of 0.80, RMSE of 0.44, and MAE of 0.30, indicating stable prediction accuracy but slightly lacking in performance. The Lasso model, with an ${\text{R}}^{2}$ of 0.70, RMSE of 0.52, and MAE of 0.50, performs the worst among regression models due to its inability to capture complex nonlinear relationships in data. The StackBoost model outperforms others, achieving the highest metrics with an ${\text{R}}^{2}$ of 0.90, RMSE of 0.29, and MAE of 0.22, indicating superior predictive precision. The results suggest that the StackBoost model overcomes the limitations of machine learning models on complex data by stacking different algorithms. The training outcomes for all six models are illustrated in Fig 4.

## High-throughput screening and analysis of optimal samples

**Fig 5:**
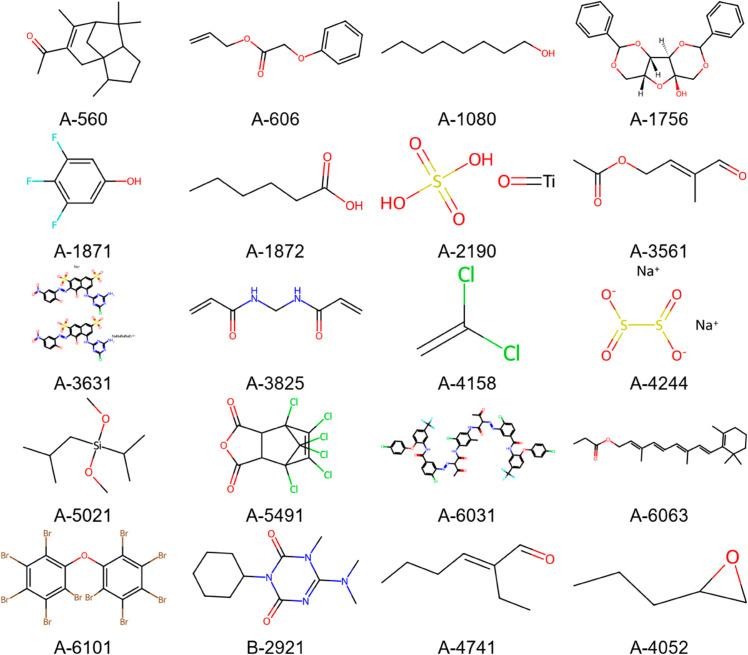
Samples with the highest solubility after high-throughput screening.

This section utilizes the feature-optimized StackBoost model to perform theoretical calculations and sample selection on a dataset of 9936 compounds, calculating the feasibility scores for each sample, as shown in Table 4. The top 20 samples were selected and visualized using the RDKit tool, with Fig 5 displaying the visualization of these 20 samples. The results indicate that samples with unique functional groups and stereo structures significantly influence solubility. Functional groups such as fluorine, chlorine, bromine, and hydroxyl enhance solubility by intensifying interactions with water molecules within the molecule. Samples with stereo structures improve solubility through their spatial interactions with water molecules. This highlights that the functional groups and molecular weight within the molecular structure are key factors affecting solubility.

**Table 4:** Synthetic feasibility scores of 20 molecular fragments.

ID	Standardized Feasibility Score	Combined Feasibility Score
A-560	0.999950555	0.187094705
A-606	0.999894394	0.123478506
A-1080	0.999831997	0.006211545
A-1756	0.999834931	0.950407173
A-1871	0.999769227	−0.477329518
A-1872	0.999686707	−0.421810131
A-2190	0.99966802	−0.626770964
A-3561	0.999647358	0.09217575
A-3631	0.999584709	0.333918678
A-3825	0.999584709	0.333918678
A-4158	0.999551251	0.065930262
A-4244	0.999454612	0.598593486
A-5021	0.999444477	−0.263384362
A-5491	0.999432867	0.423453906
A-6031	0.999432867	0.423453906
A-6063	0.999403629	−0.284102537
A-6101	0.999380376	0.312952526
B-2921	0.999353159	−1.079996942
A-4741	0.999344634	−1.205353523
A-4052	0.999328138	0.248713647

Ring structures significantly impact the stability, rigidity, and reactivity of molecules. For molecule A-1080, two saturated rings and three aliphatic rings enhance its stability and resistance to reactivity. Molecule A-1756, with two aromatic rings and three saturated rings, increases its electron stability and reactivity. The flexibility of a molecule is primarily related to the number of its rotatable bonds. Molecule A-606 has five rotatable bonds; its higher structural flexibility makes it adaptable to varied reaction conditions. Molecule A-560 has only one rotatable bond; its rigid structure provides higher stability in chemical reactions. The number of valence electrons is also a crucial factor in determining a molecule’s chemical reactivity. A-606, with fewer valence electrons, exhibits higher chemical activity, while A-560 shows lower reactivity due to its greater number of valence electrons. Additionally, molecular weight is a significant factor influencing the chemical properties of molecules. Variations in molecular weight affect the solubility and reactivity of samples in different solvents, with larger molecular weights typically offering greater stability and resistance to volatility.

## Feature analysis

**Fig 6:**
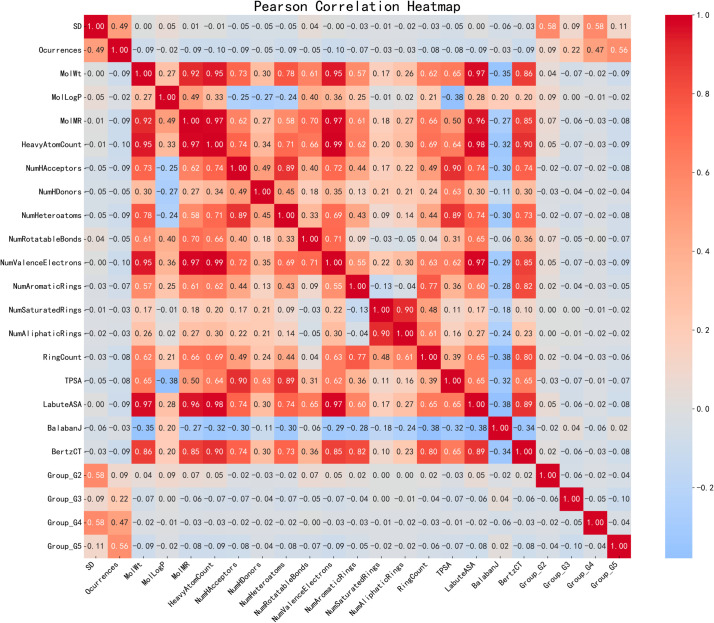
Pearson correlation analysis between pairs of feature parameters.

Calculate the Pearson correlation coefficients among 20 input features to analyze their interrelationships. As shown in Fig 6, the correlation coefficient between Mwt and MolLogP is 0.92, indicating that larger molecules typically exhibit higher solubility. There is a positive correlation of 0.87 between NumAromaticRings and NumSaturatedRings, suggesting that ring structures influence compound stability. The correlation coefficient between TPSA and LabuteASA is 0.92, indicating that surface area affects molecular solubility.

**Fig 7:**
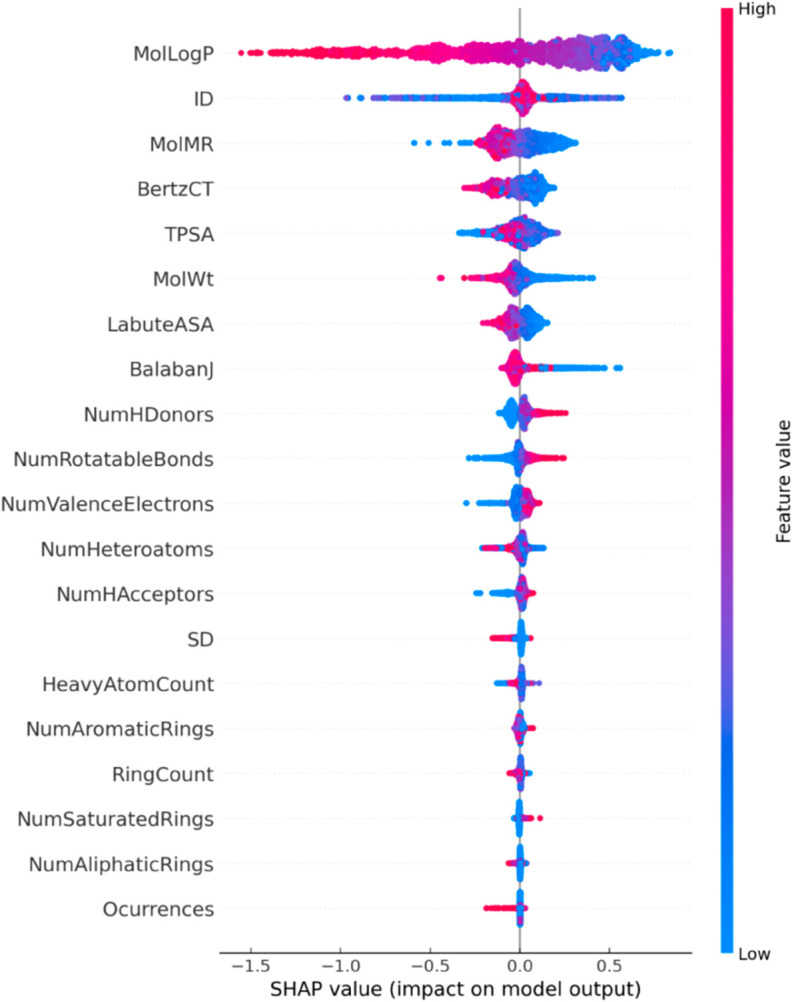
Feature importance derived from SHAP analysis.

**Fig 8:**
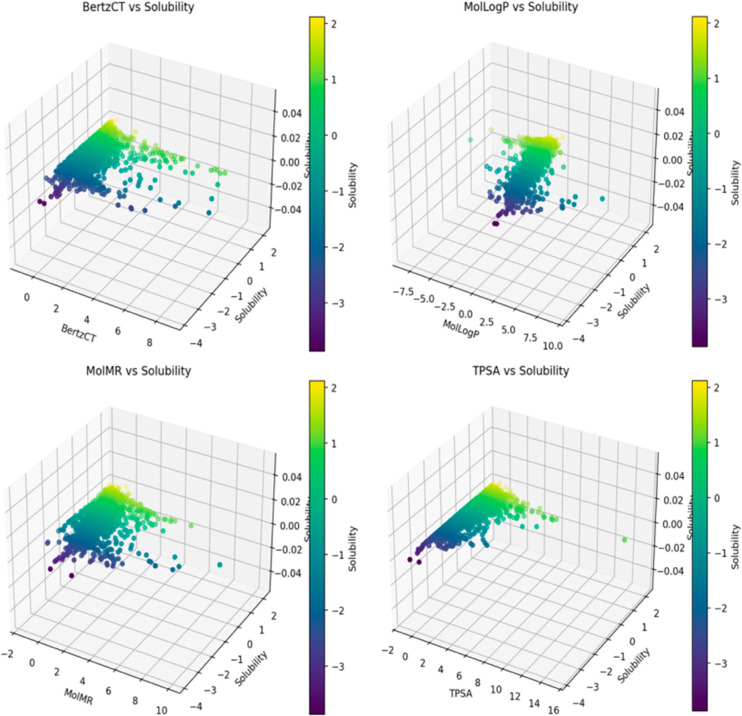
3D Scatter plot of features related to water-soluble materials.

As illustrated in Fig 7, according to SHAP analysis, MolLogP is the most influential feature with the highest SHAP value, indicating it is the most significant factor affecting compound volume. The SHAP value of MolLogP suggests a positive correlation with solubility, related to the balance between molecular hydrophilicity and hydrophobicity. The SHAP values for ID, MolMR, BertzCT, TPSA, and MolWt range from 0 to 0.5, indicating that while they correlate positively with solubility, their impact is relatively weak.SHAP values for features such as NumRotatableBonds, NumValenceElectrons, HeavyAtomCount, NumAromaticRings, and Occurrences are close to zero, suggesting they have minimal impact on solubility. Overall, SHAP analysis reveals that features like MolLogP, ID, MolMR, BertzCT, TPSA, and MolWt significantly influence model predictions, whereas NumRotatableBonds, NumValenceElectrons, NumHDonors, NumHAcceptors, HeavyAtomCount, NumAromaticRings, and Occurrences have little effect on model output. Fig 8 presents a 3D scatter plot of features MolLogP, MolMR, BertzCT, and TPSA with material solubility.

## Analysis of model transferability

**Fig 9:**
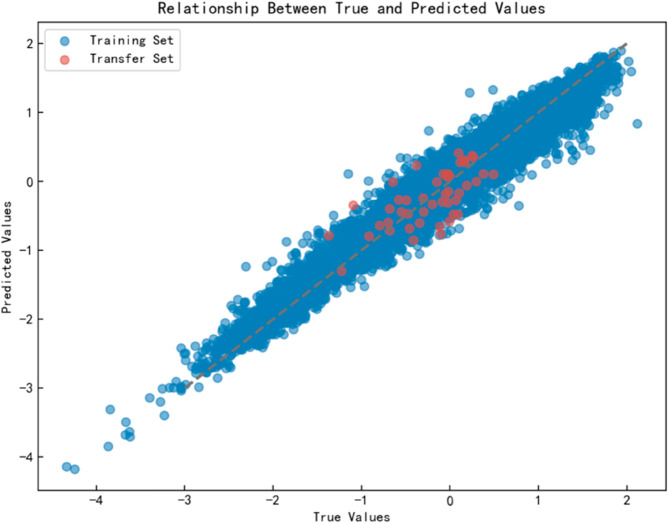
Comparison of actual vs predicted values after transfer.

To evaluate the transferability of the StackBoost model, this study trained the model using 9,936 compound samples from datasets A-H and conducted prediction and evaluation on 46 compound samples from dataset I to test the model’s transferability and generalization ability. When the model was transferred to dataset I, the test results showed an ${\text{R}}^{2}$ of 0.84, an RMSE of 0.40, and an MAE of 0.29, indicating a decrease in accuracy after transfer but still demonstrating the model’s transferability. Fig 9 presents a comparison between the true and predicted values. The figure shows that the distribution of dataset I is broader and more variable than that of datasets A-H. By standardizing the features of datasets A-H and I to the same normal distribution to eliminate differences in data distribution, the StackBoost model was retrained and evaluated, resulting in improved performance. This indicates that differences in data distribution are a significant factor affecting the model’s transfer performance, as such differences impact prediction accuracy. After standardizing the data to the same normal distribution, the model demonstrated better transferability and improved robustness in cross-domain transfer.

## Conclusion

The study used a dataset of water solubility data for 9,982 compounds from the AqSolDB database. Data preprocessing was conducted through standardization, conversion to SMILES representation, and One-Hot encoding, followed by hyperparameter tuning using Optuna. In this research, five ensemble models—AdaBoost, GBRT, LGBM, XGBoost, and RF—were employed to train and predict the dataset. Additionally, a StackBoost model was built, which not only accurately analyzed the relationships between features but also improved the model’s predictive accuracy. The StackBoost model achieved an ${\text{R}}^{2}$ of 0.90, an RMSE of 0.29, and an MAE of 0.22, demonstrating strong predictive ability.

To further validate the practicality of the StackBoost model, high-throughput screening of 9,936 samples was conducted, yielding feasibility scores for each sample and identifying compounds with high water solubility. The screening results indicated that the StackBoost model could effectively identify compounds with high water solubility, confirming its effectiveness and reliability in recognizing the water solubility of compounds. The innovation of the StackBoost model lies in its ability to overcome the limitations of single models in handling complex relationships by stacking multiple machine learning models. Furthermore, hyperparameter tuning using Optuna enhanced the model’s stability and predictive accuracy. The integration of Bayesian optimization techniques for hyperparameter adjustments effectively reduced the risk of overfitting and strengthened the model’s robustness. Although the model’s ${\text{R}}^{2}$ value decreased from 0.90 to 0.8362 on Dataset I, it still demonstrated its transferability. The application of the StackBoost model for high-throughput screening of 9,936 samples yielded feasibility scores and identified high water solubility compounds, indicating that the StackBoost model successfully overcame the limitations of single models in capturing complex relationships, enhancing predictive accuracy and stability.

Despite demonstrating good performance, there remains room for further optimization of the StackBoost model. Future research could explore more effective ways to handle high-dimensional feature data to improve predictive accuracy. Additionally, taking into account the diversity of datasets and variations in experimental design, future work should include a broader range of datasets and various types of compounds to validate the model’s generalization capabilities. Furthermore, this study has several limitations: first, the size of the dataset used may not be sufficient to comprehensively cover all possible compound types; thus, future efforts should consider more diversified sample datasets. Second, the computational cost of the StackBoost model is relatively high, especially when dealing with large amounts of compound data, and future optimizations for computational efficiency may be necessary. Based on these research findings, future work should focus on the following aspects: optimizing the computational efficiency of the StackBoost model to make it suitable for larger-scale compound screening; further enhancing the model’s stability and accuracy when handling high-dimensional data; and attempting to integrate other datasets and domain-specific knowledge to improve the model’s generalization ability and cross-domain applications.
